# Prenatal alcohol exposure and infant gross motor development: a prospective cohort study

**DOI:** 10.1186/s12887-019-1516-5

**Published:** 2019-05-14

**Authors:** Delyse Hutchinson, George J. Youssef, Clare McCormack, Judy Wilson, Steve Allsop, Jake Najman, Elizabeth Elliott, Lucinda Burns, Sue Jacobs, Ingrid Honan, Larissa Rossen, Hannah Fiedler, Samantha Teague, Joanne Ryan, Craig A. Olsson, Richard P. Mattick

**Affiliations:** 1 National Drug and Alcohol Research Centre, University of New South Wales, Sydney, Australia; 2 Faculty of Health, School of Psychology, Centre for Social and Early Emotional Development, Deakin University, Geelong, Victoria 3125 Australia; 3 Murdoch Children’s Research Institute, Centre for Adolescent Health, Royal Children’s Hospital, Melbourne, Australia; 4 Department of Paediatrics, University of Melbourne, Royal Children’s Hospital, Melbourne, Australia; 5 National Drug Research Institute, Curtin University, Perth, Australia; 6 Queensland Alcohol and Drug Research and Education Centre and Schools of Public Health and Social Science, University of Queensland, Brisbane, Australia; 7 Discipline of Child and Adolescent Health, Faculty of Medicine and Health, University of Sydney, Sydney, Australia; 8 Sydney Children´s Hospitals Network, Westmead, Sydney, Australia; 9 Department of Obstetrics, Royal Prince Alfred Hospital, Sydney, Australia; 10 School of Psychological Sciences, University of Melbourne, Melbourne, Victoria Australia

**Keywords:** Alcohol, Motor Skills, Infancy, Perinatal

## Abstract

**Background:**

Maternal alcohol consumption in pregnancy may have adverse effects on child gross motor (GM) development. There have been few human studies on this topic, particularly ones examining low exposure. This study examined the association between prenatal alcohol exposure (PAE) and infant GM development at 12-months of age.

**Methods:**

Participants were 1324 women recruited from antenatal clinics in Sydney and Perth, Australia. Maternal and paternal alcohol use was assessed in pregnancy via interview; offspring GM development was measured at 12-months with the Bayley Scales of Infant Development (BSID-III).

**Results:**

Any alcohol use in pregnancy was common: 56.1%, of pregnant women drank early in Trimester one (0–6 weeks), however this reduced to 27.9% on average thereafter and at predominantly low levels. However, infant BSID GM scale scores were not found to differ significantly as a function of PAE in the first 6-weeks (low, moderate, binge or heavy PAE), nor with low PAE across pregnancy.

**Conclusions:**

We found no evidence to suggest that low PAE is associated with measurable impairment in infant GM development at 12-months. Further research is needed to examine potential PAE impacts on GM development in heavier exposure groups and through the childhood years when subtle GM deficits may be more detectable.

**Electronic supplementary material:**

The online version of this article (10.1186/s12887-019-1516-5) contains supplementary material, which is available to authorized users.

## Background

Prenatal alcohol exposure (PAE) has been associated with impairments in infant motor development [1, 2]. Infants diagnosed with Fetal Alcohol Spectrum Disorders (FASD) may exhibit a range of motor impairments [3], including orthopedic and structural deficits, tapering of the distal phalanges, decreased elbow pronation/supination, clubfoot and hand tremors [4–8]. Neuroimaging studies have identified damage to specific regions of the brain among individuals with PAE or FASD [9–13]. In animal studies, PAE has been associated with impaired spinal and peripheral nerve myelination [14, 15], and with reduced motor coordination, speed, response, reflexes, activity, and tone [16, 17].

A number of systematic reviews have examined the relationship between PAE and motor skills. A 2011 review found that high levels of PAE (10 to 30 drinks per week) were associated with impaired offspring motor function [1]. The review did not examine whether specific types of motor skills were more likely to be affected; nor whether gross motor (GM) skills (i.e., coordination of movement using the large muscles of the body) were affected independently of fine motor (FM) skills (i.e., precise, coordinated movements).

A recent review found that neither mild (≤3 drinks per week), moderate (≤6 drinks per week, including some women who drank at least 3 drinks per week), nor binge (≥4 or ≥ 5 drinks per occasion) PAE were significantly associated with motor impairment in children below 5 years of age [18]. Again, however, this study did not differentiate GM from FM skills. Distinguishing these skills and their associations with PAE is important because management strategies to address GM deficits differ considerably from FM interventions [19].

In 2014 Lucas et al. [2] published a systematic review and meta-analysis of GM impairment in children (mean age 3 days to 13 years) diagnosed with FASD or exposed to moderate (2 to ≥14 drinks per week) to heavy (> 10 to 28 drinks per week) or binge (≥5 drinks per occasion) PAE. Results indicated that children with FASD were three times more likely to have a GM impairment, yet moderate to heavy and binge PAE were not significantly associated with GM impairment. Notably, this review did not examine low PAE compared with no PAE [2]. Evidence for GM impairment has not yet been established following only low PAE [20, 21].

This study aimed to examine GM skills in a cohort of 1324 infants (mean age = 12.20 months) from a longitudinal pregnancy cohort with multi-wave data on both the *timing* and *dose* of PAE. Uniquely, PAE was assessed at four time points through pregnancy: *Trimester one 0–6 weeks* (T1a); *Trimester one 7–12 weeks* (T1b); *Trimester two, 13–27 weeks* (T2); *Trimester three, 28 weeks until birth* (T3). GM skills were assessed using the Bayley Scales of Infant Development (BSID-III) [22] at 12-months infant age. Specifically, the aims of the study were threefold: (1) examine the frequency and quantity of maternal alcohol use across pregnancy; (2) examine maternal, infant and partner characteristics associated with PAE in pregnancy compared to abstainers; and, (3) determine whether GM skills were impaired among infants after PAE compared with infants of abstainers, accounting for potential maternal, paternal and infant confounders.

## Methods

### Participants

Data were from the Triple B Pregnancy Cohort Study, a prospective Australian study of 1634 pregnant women recruited in 2009–13 at antenatal clinics in NSW (*n* = 1305) and WA (*n* = 318) [23]. Ethical approval was granted by university and hospital Human Research Ethics Committees. Eligibility criteria included: pregnancy; aged ≥16 years; no major medical complications (mother/fetus); mother/both parents the primary caregiver/s; mentally able to complete assessments; English literary; and informed consent.

Data were collected in pregnancy at T1a, T1b, T2, T3, and at 8-weeks, and 12-months post-birth. By separating T1 into two periods the effects of early PAE could be examined. Most women reported pregnancy awareness around 5–6 weeks gestation [24]; as such, a six-week split broadly represents pre- and post-pregnancy awareness. Of the participants with GM outcome data, we excluded women with inconsistent drinking behaviour (*n* = 141) and who had infants with very low birth weight (< 1.6 kg; n = 1). For women who gave birth to twins/triplets (*n* = 34), one child was selected at random for analysis. The final sample comprised 1324 participants (755 with participating partners).

### Measures

Study measures are described in Table 1.

**Table 1:** Summary of Exposure, Outcome and Other Background Confounding and Precision Measures

Construct	Measurement information and/or exemplar item	Response categories	Participant/timepoint	Source (additional information)
Exposure variable: Maternal alcohol use	Quantity and frequency of maternal alcohol use (standard drink = 10 g of alcohol).	1. Abstinent: no consumption; 2. Low: ≤7 standard drinks per week, up to 2 standard drinks per occasion; 3. Moderate: ≤7 standard drinks per week, > 2 to ≤4 standard drinks per occasion; 4. Binge: ≤7 standard drinks per week, > 4 standard drinks per occasion^a^; 5. Heavy: > 7 standard drinks per week; at least weekly or more.	Mother (T1-T3)	O’Leary et al.’s (2010) Prenatal Alcohol Exposure (PAE) categories
Outcome variable: Infant Gross Motor (GM) Development	Example: Infant can sit upright unsupported.	Standardised GM scaled scores (mean = 10, SD = 3), adjusted for age and prematurity.	Infant (12 M)	Bayley Scales of Infant and Toddler Development - Third edition (BSID-III) [22]
Background confounder and precision variables: Age at birth	Calculated based on mother/partner and infant date of birth.	≤ 24 years; 25–29 years; 30–35 years; ≥ 36 years.	Mother, partner (T3/8W)	
Education	What is your highest level of education?	Less than Year 12; Year 12; Trade certificate, diploma or apprenticeship; Tertiary qualification.	Mother, partner (T3)	
Birth country	In which country were you born?	Australia; other English-speaking country; non-English-speaking country.	Mother, partner (T3)	
Single parent household	What is your current marital status?	Single parent household (Yes or No).	Mother (T3)	
Aboriginal and Torres Strait Islander status	Are you Aboriginal and/or Torres Strait Islander?	Yes or No.	Mother, partner (T3)	
First language spoken	What was your first language spoken?	English; Language other than English.	Mother, partner (T3)	
Socio-economic status (SES)	The Socio-Economic Indexes for Areas (SEIFA) data package was used to classify participants into low, moderate or high socio-economic status (SES) deciles based on residential postcode.	Low = deciles 1–3; Moderate = deciles 4–7; High = deciles 8–10.	Mother, partner (T3)	SEIFA data package [45]
State of residence	State of residence in Australia	New South Wales (NSW) or Western Australia (WA).	Mother (T3)	
Tobacco in pregnancy	Tobacco use in pregnancy (ever used).	Yes or No.	Mother (T1-T3), partner (T3)	
Illicit substance in pregnancy	Illicit substance use in pregnancy (ever used).	Yes or No.	Mother (T1-T3), partner (T3)	
Maternal Depression	In the last 7 days …. I have felt sad or miserable.	Yes most of the time; Yes quite often; Not very often; No not at all. Coded as Normal (scores <9) or Elevated (scores ≤9).	Mother (T1-T3), partner (T3)	Edinburgh Depression Scale (EDS) [26]
Stress	Over the past week …. I found it hard to wind down.	Not at all; Some degree, or some of the time; Considerable degree, or a good part of the time; Very much, or most of the time. Coded as Normal (scores <10) or Elevated (scores ≤10).	Mother (T1-T3), partner (T3)	Depression, Anxiety and Stress Scales [27]
Anxiety	Over the past week …. I felt I was close to panic.	Not at all; Some degree, or some of the time; Considerable degree, or a good part of the time; Very much, or most of the time. Coded as Normal (scores <19) or Elevated (scores ≤19).	Mother (T1-T3), partner (T3)	Depression, Anxiety and Stress Scales [27]
Victim of spousal abuse	My partner insults or shames me in front of others.	Never; Rarely; Occasionally; Frequently; Very Frequently Scores indicating at least moderate severity on the physical or non-physical subscales were combined then binary coded to indicate the presence or absence of abuse (Yes or No).	Mother, partner (T3)	The Index of Spousal Abuse [28]
Estimated IQ	TOPF scores were converted to provide a valid predictor of the full-scale WAIS-IV IQ [46].	Low average = ≤84; Average = 85–99; High average = 100–114; Superior = ≥115.	Mother, partner (12 M)^b^	Test of Premorbid Functioning (TOPF) [29]
Parity	Total number of pregnancies carried to term prior to the current pregnancy [47].	0; 1–2; ≥3.	Mother (T3)	
Pre-pregnancy body mass index (BMI)	BMI was calculated based on self-reported pre-pregnancy weight and height [48].	Underweight = < 18.49; Normal weight = 18.50–24.99; Overweight = 25.00–29.99; Obese = ≥30.00 [49].	Mother, partner (T3)	
Pregnancy planning	How did you feel about becoming pregnant?	I wanted to become pregnant; I didn’t want to become pregnant; I hadn’t thought about becoming pregnant; Other. Responses were re-coded as pregnancy planned versus not planned; ‘other’ responses were classified based on examination of open-ended responses (*n* = 69).	Mother (T3)	
Partner alcohol use	Quantity and frequency of partner alcohol use (standard drink = 10 g of alcohol)	1. Abstinent: no consumption; 2. Low: ≤7 standard drinks per week, up to 2 standard drinks per occasion; 3. Moderate: < 14 standard drinks per week, > 2 to ≤4 standard drinks per occasion; 4. Binge: ≤14 standard drinks per week, > 4 standard drinks on one occasion; 5. Heavy: > 14 standard drinks per week; at least weekly consumption or more.	Partner (T3)	Australian NHMRC guidelines for drinking among non-pregnant adults were used to classify binge and heavy drinking [50]
Infant sex	What gender is your baby?	Female; Male.	Mother (8 W)	Extracted from hospital records (*Blue Books)*, as recorded by hospital staff at birth.
Prematurity	Number of weeks gestation at birth.	Not premature = ≥37 weeks; Premature = ≤36 weeks.	Mother (8 W)	Extracted from hospital records (*Blue Books)*, as recorded by hospital staff at birth.
Birthweight	Weight in grams at birth, coded into two categories based on Australian norms [51].	Normal= > 10th percentile for gestational age; Small for gestational age (SGA) = ≤10th percentile for gestational age.	Mother (8 W)	Extracted from hospital records (*Blue Books)*, as recorded by hospital staff at birth.
Head circumference	Reported in centimetres.	Normal= > 3rd percentile; Small = ≤3rd percentile.	Mother (8 W)	Extracted from hospital records (*Blue Books)*, as recorded by hospital staff at birth.
Apgar score (5 min)	Post-birth measure of infant health.	Normal = ≥7; Problems at birth = < 7.	Mother (8 W)	Extracted from hospital records (*Blue Books)*, as recorded by hospital staff at birth.

^a^The definition of *binge* was altered to be more consistent with the Australian National Health and Medical Research Council (NHMRC) guideline around risky drinking, and refers to heavy episodic drinking [24, 50]. ^b^A small proportion of parent TOPF data was collected at infant age 3-years as part of a preschool nested study

#### Alcohol use

Maternal drinking was assessed via interview. Self-reported frequency and quantity (10 g of alcohol per standard drink) of typical use during each trimester, and occasions when women drank more, were recorded. Alcohol use during T3 was assessed retrospectively at the 8-week interview so that consumption across the trimester was captured. Average weekly alcohol consumption was calculated using O’Leary et al.’s PAE categories: *abstinent, low, moderate, binge* and *heavy* [25]. A subsample of 85 participants was randomly selected for urine analysis in T3 to confirm self-reported illicit substance use. Agreement between self-reported substance use and urine analysis was 97%, indicating that the information provided via interview was reliable.

#### Infant GM development

The BSID-III was administered to children at 12-months (mean age = 12.20 months, SD = 0.86, range = 8 to 22 months) by qualified assessors in participants’ homes [22]. Inter-rater reliability in a randomly selected sub-sample was high (Cronbach’s alpha = 0.99; *n* = 27). Infant scores on the BSID-III were age adjusted for prematurity.

#### Potential confounders

*Maternal socio-demographic background factors* included: maternal age at birth; education; birth country; single parent status; Aboriginal and Torres Strait Islander descent; language and household socio-economic status (SES).

*Precision variables:* To further isolate a causal role for any observed association between PAE and offspring GM development, we systematically entered a range of other possible determinants into the multivariate models. These included: *maternal substance use, physical and mental health in pregnancy* (tobacco/illicit drug use; depression, anxiety and stress [26, 27]; spousal abuse [28]), *estimated IQ* [29], *parity*, *pre-pregnancy body mass index* (BMI), *pregnancy planning,* and *infant sex* and *birth outcomes* (prematurity, birthweight, head circumference, Apgar score). Potential partner-related confounding factors assessed at T3 were also entered in a series of supplementary analyses. Partner data were available for 57% (*n* = 754) of participating mothers.

### Planned analyses

Analyses were conducted using STATA 14 and SPSS 20 [30, 31]. Missing data was accounted for using multiple imputation [32, 33]. There were four stages of analysis. First, we described maternal drinking patterns (low, moderate, binge and heavy drinking versus abstinence) at all pregnancy timepoints (T1a, T1b, T2 and T3), and partner drinking patterns at T3. Second, binary logistic regression models estimated the association between mother, infant and partner-related factors and PAE (i.e., any drinking in pregnancy versus abstinence).

In the third stage, logistic regression analyses examined the relationship between PAE (T1a, T1b, T2 and T3) and infant GM outcomes at 12-months, controlling for background socio-demographics, and other potential maternal, infant and partner confounders. At T1a all PAE categories were examined. However, due to the low frequency of moderate, binge and heavy drinking in the sample for T1b, T2 and T3 (see Table 2), only low PAE was examined. The reference category was abstinence. The primary outcome was the BSID-III scaled GM score. The unadjusted results were examined first, followed by increasing levels of adjustment for potential confounders. The adjusted analyses included the maternal socio-demographic background factors, followed by factors found to be associated with maternal drinking at the univariate level (*p* < .10) [34].

**Table 2:** Patterns of Alcohol Use by Mothers across Pregnancy (*N* = 1324)

	Alcohol use category				
	Abstinent	Low (≤7 drinks per week, up to 2 per occasion)	Moderate (≤7 drinks per week, > 2 to ≤4 per occasion)	Binge (≤7 drinks per week, > 4 per occasion)	Heavy (> 7 drinks per week, weekly or more)
Trimester 1a (first 6 weeks)					
*n* (%)	514 (38.8)	291 (22.0)	55 (4.2)	211 (15.9)	184 (14.0)
Drinking days per week, M (SD)	0	1.09 (1.10)	0.91 (0.60)	0.98 (0.87)	3.73 (1.83)
Typical grams consumed per occasion, M (SD)	0	12.70 (4.11)	28.31 (4.30)	38.22 (29.33)	50.74 (54.01)
Typical grams consumed per week, M (SD)	0	17.50 (17.70)	27.50 (18.55)	30.81 (21.51)	181.16 (200.02)
Trimester 1b (second 6 weeks)					
*n* (%)	943 (71.2)	224 (16.9)	24 (1.8)	41 (3.1)	24 (1.8)
Drinking days per week, M (SD)	0	0.58 (0.63)	0.66 (0.64)	0.80 (0.67)	3.62 (1.80)
Typical grams consumed per occasion, M (SD)	0	11.13 (4.81)	29.44 (4.16)	28.21 (20.07)	74.36 (121.52)
Typical grams consumed per week, M (SD)	0	8.32 (9.11)	18.74 (19.15)	32.43 (23.56)	27.18 (43.05)
Trimester 2					
*n* (%)	894 (67.5)	339 (25.6)	37 (2.8)	14 (1.1)	13 (1.0)
Drinking days per week, M (SD)	0	0.70 (0.72)	0.80 (0.65)	1.23 (0.90)	4.40 (2.10)
Typical grams consumed per occasion, M (SD)	0	12.46 (3.82)	29.64 (3.45)	25.14 (14.77)	58.23 (75.12)
Typical grams consumed per week, M (SD)	0	9.04 (10.39)	23.42 (20.28)	34.39 (21.65)	189.72 (223.25)
Trimester 3					
*n* (%)	902 (68.1)	337 (25.5)	31 (2.3)	10 (0.8)	13 (1.0)
Drinking days per week, M (SD)	0	1.30 (0.40)	2.93 (0.32)	3.30 (16.20)	2.31 (1.50)
Typical grams consumed per occasion, M (SD)	0	12.82 (3.60)	29.27 (3.19)	32.50 (1.62)	23.08 (14.51)
Typical grams consumed per week, M (SD)	0	11.89 (12.90)	25.38 (18.58)	29.06 (25.04)	111.92 (34.97)

Note: (%) values do not sum to 100% due to missing data. *Standard drink* = 10 g of alcohol

Finally we examined whether the effect of PAE may differ according to an individual’s risk of being exposed to alcohol based on their baseline characteristics [35]. To do this we calculated the propensity of a woman to consume alcohol at low-levels. We then stratified the sample into groups indicating higher and lower risk of low-level alcohol exposure based on their propensity score and compared infant GM outcomes between these groups (see Additional file 2: Section B for a detailed description).

## Results

### Patterns of alcohol use by mothers across pregnancy

Most women reported alcohol use at some point in pregnancy (61.2%; Table 2). Among T1a drinkers, low-level use was most frequently endorsed (22.0%), followed by binge (15.9%) and heavy drinking (14.0%), respectively. There was a marked change in drinking patterns in T1b. Notably, abstinence increased from 38.8 to 71.2%, and of those who did report drinking alcohol, most did so at low-levels (16.9%). Binge and heavy drinking decreased to 3.1 and 1.8%, respectively. This trend remained consistent through T2 and T3, although some women did return to low-level drinking as their pregnancy progressed (low-level drinkers: T2, 25.6%; T3, 25.5%).

### Patterns of alcohol use by partners

Additional file 1: Table S1 shows the pattern of alcohol use reported by partners. Among those who drank (85.6%), binge drinking was most common (27.9%), followed by low (20.3%), moderate (14.4%) and heavy drinking (13.3%), respectively.

### Characteristics associated with maternal drinking in pregnancy

Univariate tests compared whether abstainers and pregnancy drinkers (at any level) differed on background socio-demographics, other substance use, and physical and psychological factors (Table 3). The results show that, relative to abstainers, women who drank alcohol had greater odds of being older (e.g., 30–35 years, 1.97, 95% CI, 1.20–3.24); completing high school (2.61, 95% CI, 1.48–4.61); having moderate (2.29, 95% CI, 1.31–4.02) or high SES (4.42, 95% CI, 2.56–7.64); being born in an English speaking country (1.88, 95% CI, 1.33–2.66); living in a household with multiple parents (single parent: 0.61, 95% CI, 0.39–0.95); and speaking English as their first language (2.34, 95% CI, 1.77–3.09); and lower odds of living in a single parent household (0.61, 95% CI, 0.39-0.95). Other factors associated with pregnancy drinking included: smoking in pregnancy (1.67, 95% CI, 1.18–2.36); reduced anxiety (0.76, 95% CI, 0.57–0.99); and higher estimated IQ (e.g., a score of 100–114, 3.02, 95% CI, 2.01–4.53).

**Table 3:** Maternal and Infant Factors Associated with Alcohol Use (Pooled Data, *N* = 1324)

		Abstainers (*n* = 452) *n* (column %)	Drinkers (*n* = 872) *n* (column %)	Drinkers vs abstainers - Unadjusted OR (95% CI)
Maternal factors				
Age	≤ 24	34 (7.5)	37 (4.3)	Ref
	25–29	112 (24.7)	162 (18.6)	1.33 (0.78–2.25)
	30–35	185 (41)	400 (45.9)	1.97 (1.2–3.24)**
	≥ 36	121 (26.7)	273 (31.2)	2.06 (1.23–3.45)**
Level of education	Less than Year 12	44 (9.7)	35 (4.1)	Ref
	Year 12	46 (10.2)	97 (11.1)	2.61 (1.48–4.61)**
	Certificate / Diploma	67 (14.8)	127 (14.5)	2.34 (1.37–4)**
	Bachelor or higher	295 (65.3)	613 (70.3)	2.57 (1.61–4.11)***
Household SES	Low	38 (8.4)	22 (2.5)	Ref
	Moderate	172 (38.1)	229 (26.3)	2.29 (1.31–4.02)**
	High	242 (53.5)	621 (71.2)	4.42 (2.56–7.64)***
State of Residence	New South Wales	378 (83.8)	739 (84.7)	Ref
	Western Australia	73 (16.2)	134 (15.3)	0.94 (0.69–1.29)
Country of birth	Australia	248 (54.8)	512 (58.7)	Ref
	Other English speaking	50 (11.1)	194 (22.3)	1.88 (1.33–2.66)***
	NESB	154 (34.1)	166 (19)	0.52 (0.4–0.68)***
Single parent household	No	412 (91.3)	825 (94.5)	Ref
	Yes	39 (8.7)	48 (5.5)	0.61 (0.39–0.95)*
Aboriginal or Torres Strait Islander	No	441 (97.6)	861 (98.7)	Ref
	Yes	11 (2.4)	11 (1.3)	0.53 (0.22–1.24)
English first language	No	166 (36.9)	174 (20)	Ref
	Yes	285 (63.1)	698 (80)	2.34 (1.77–3.09)***
Tobacco in pregnancy	No	402 (88.9)	722 (82.8)	Ref
	Yes	50 (11.1)	150 (17.2)	1.67 (1.18–2.36)**
Illicit substances ever in pregnancy	No	434 (96.2)	818 (93.7)	Ref
	Yes	17 (3.8)	55 (6.3)	1.68 (0.96–2.95)
Depression	Normal	321 (71.1)	626 (71.8)	Ref
	Elevated	130 (28.9)	246 (28.2)	0.97 (0.75–1.25)
Anxiety	Normal	328 (72.7)	679 (77.9)	Ref
	Elevated	123 (27.3)	193 (22.1)	0.76 (0.57–0.99)*
Stress	Normal	377 (83.5)	711 (81.5)	Ref
	Elevated	75 (16.5)	161 (18.5)	1.15 (0.83–1.59)
Victim of spousal abuse	No	431 (95.5)	841 (96.4)	Ref
	Yes	20 (4.5)	31 (3.6)	0.79 (0.42–1.48)
Estimated IQ (All participants)	≤ 84	106 (23.4)	88 (10)	Ref
	85–99	167 (37)	301 (34.5)	2.17 (1.47–3.22)***
	100–114	135 (29.9)	337 (38.6)	3.02 (2.01–4.53)***
	≥ 115	44 (9.7)	147 (16.8)	4.06 (2.34–7.03)***
Estimated IQ (Native English only *n* = 968)	≤ 84	42 (14.9)	50 (7.2)	Ref
	85–99	98 (34.8)	217 (31.6)	1.86 (1.05–3.3)*
	100–114	102 (36.3)	284 (41.4)	2.33 (1.36–4.01)**
	≥ 115	39 (14.1)	136 (19.8)	2.9 (1.47–5.73)**
Parity	0	239 (53)	515 (59.1)	Ref
	1-Feb	187 (41.5)	324 (37.1)	0.8 (0.63–1.02)
	3+	25 (5.5)	33 (3.8)	0.62 (0.36–1.07)
Body Mass Index	Underweight	11 (2.5)	10 (1.1)	0.43 (0.18–1.04)
	Normal weight	174 (38.5)	346 (39.6)	Ref
	Overweight	107 (23.8)	278 (31.8)	1.3 (0.97–1.74)
	Obese	159 (35.1)	239 (27.4)	0.76 (0.58–1.00)
Pregnancy planning	Planned	373 (82.7)	715 (82)	Ref
	Unplanned	78 (17.3)	157 (18)	1.05 (0.77–1.42)
Infant factors				
Baby sex	Male	230 (51)	461 (52.9)	Ref
	Female	221 (49)	411 (47.1)	0.93 (0.74–1.17)
Gestational age	Not preterm (37+ weeks)	418 (92.6)	839 (96.2)	Ref
	Preterm (<=36 weeks)	33 (7.4)	33 (3.8)	0.5 (0.3–0.81)**
Birthweight	Not small (>10th percentile)	396 (87.8)	786 (90.1)	Ref
	Small (<= 10th percentile)	55 (12.2)	86 (9.9)	0.79 (0.55–1.13)
Head circumference	Not small	426 (94.4)	837 (96)	Ref
	Small (<3rd percentile)	25 (5.6)	35 (4)	0.71 (0.41–1.23)
Apgar score (at 5mins)	> = 7	444 (98.4)	854 (97.9)	Ref
	<7	7 (1.6)	18 (2.1)	1.31 (0.45–3.83)

*p < 0.05; **p < 0.01, ***p < 0.001

Univariate tests also compared whether infants of abstainers and pregnancy drinkers (at any level) differed on sex and birth indicators (Table 3). Compared to infants of abstainers, infants born to mothers who drank in pregnancy were less likely to be born preterm (< 36 weeks gestation; 0.05, 95% CI, 0.3–0.82). No significant differences were found in sex, birthweight, head circumference or Apgar scores.

### Characteristics associated with paternal drinking

Additional file 1: Table S2 shows the results of univariate tests comparing the characteristics of partners of abstainers with partners of pregnancy drinkers. Compared to partners of abstainers, partners of drinkers had three-fold greater odds of being low-level drinkers (2.99, 95% CI, 1.79–5.00); four-fold greater odds of being moderate drinkers (4.27, 95% CI, 2.40–7.63); six-fold greater odds of being binge drinkers (6.08, 95% CI, 3.65–10.12); and almost eight-fold greater odds of being heavy drinkers (7.78, 95% CI, 4.06–14.91). Partners of drinkers were older (e.g., 30–35 years, 2.89 95% CI, 1.52–5.52), less likely to report a non-English speaking background (0.49, 95% CI, 0.34–0.72); and more likely to report English as their first language (2.08, 95% CI, 1.40–3.11), compared to partners of abstainers.

### PAE and infant GM development

Regression analyses were used to examine the relationship between PAE exposure and infant GM development at 12-months (Table 4).

**Table 4:** Regression Results For Maternal Alcohol Use and Infant Gross Motor Outcomes (Pooled Data, *N* = 1324)

	Unadjusted b (95%CI)	Adjusted for maternal^a^ b (95%CI)	Adjusted for maternal^b^ b (95%CI)	Adjusted for maternal^c^ b (95%CI)	Adjusted for maternal + infant^d^ b (95%CI)
Trimester 1a (first 6 weeks) (*n* = 1324)					
Abstinent	Ref	Ref	Ref	Ref	Ref
Low (≤7 drinks per week, up to 2 per occasion)	−0.29 (−0.68–0.11)	−0.35 (−0.75–0.05)	−0.35 (−0.74–0.05)	−0.37 (−0.78–0.03)	−0.39 (−0.79–0.01)
Moderate (≤7 drinks per week, > 2 to ≤4 per occasion)	− 0.49 (−1.24–0.27)	− 0.64 (−1.39–0.12)	− 0.63 (−1.39–0.12)	− 0.62 (−1.38–0.14)	− 0.66 (−1.42–0.1)
Binge (≤7 drinks per week, > 4 per occasion)	− 0.23 (− 0.66–0.19)	−0.32 (− 0.75–0.12)	−0.31 (− 0.75–0.13)	−0.29 (− 0.74–0.15)	−0.3 (− 0.74–0.15)
Heavy (> 7 drinks per week, weekly or more)	0.02 (− 0.44–0.48)	− 0.04 (− 0.5–0.42)	− 0.02 (− 0.5–0.45)	−0.02 (− 0.5–0.46)	−0.06 (− 0.54–0.42)
Trimester 1b* (second 6 weeks) (*n* = 1227)					
Abstinent	Ref	Ref	Ref	Ref	Ref
Low (≤7 drinks per week, up to 2 per occasion)	−0.23 (− 0.63–0.17)	−0.31 (− 0.71–0.09)	−0.3 (− 0.71–0.1)	−0.31 (− 0.72–0.1)	−0.32 (− 0.73–0.09)
Trimester 2* (*n* = 1259)					
Abstinent	Ref	Ref	Ref	Ref	Ref
Low (≤7 drinks per week, up to 2 per occasion)	−0.02 (− 0.35–0.32)	0.03 (− 0.32–0.37)	0.03 (− 0.32–0.38)	0.02 (− 0.33–0.37)	− 0.01 (− 0.36–0.34)
Trimester 3* (*n* = 1270)					
Abstinent	Ref	Ref	Ref	Ref	Ref
Low (≤7 drinks per week, up to 2 per occasion)	−0.12 (− 0.46–0.21)	−0.03 (− 0.37–0.31)	−0.03 (− 0.38–0.31)	−0.04 (− 0.4–0.31)	−0.08 (− 0.43–0.28)

Note: *Standard drink* = 10 g of alcohol. *Moderate, *Binge* and *Heavy* categories were not assessed after T1a due to infrequent reporting of these drinking patterns in the sample

^a^Adjusted for Mother-related background variables (Age at birth, Education, SEIFA, State of residence, Country of birth, Single parent household, Aboriginal and Torres Strait Islander status, Native language)

^b^Adjusted for Mother-related background variables + Substance use variables (Pregnancy smoked, Pregnancy illicit drugs)

^c^Adjusted for Mother-related background variables + Physical and psychological variables (Pregnancy Anxiety, IQ, Parity, BMI)

^d^Adjusted for all previous Mother-related variables + Infant-related variables (Gestational age)

For T1a, in the unadjusted analyses, there were no significant associations between PAE and GM outcomes. This relationship remained unchanged after adjustment. For T1b, T2 and T3, the results were consistent when PAE exposure was binary (i.e., abstinence versus low-level drinking); namely, low PAE was not significantly associated with GM development in infants at 12-months in the unadjusted, nor in the adjusted analyses. Table 5 shows the marginal means and 95% CIs for PAE and GM outcomes at all levels of adjustment.

**Table 5:** Marginal means for maternal alcohol use and infant gross motor outcomes (pooled data, *N* = 1324)

	Unadjusted M (95%CI)	Adjusted for maternal^a^ M (95%CI)	Adjusted for maternal^b^ M (95%CI)	Adjusted for maternal^c^ M (95%CI)	Adjusted for maternal + infant^d^ M (95%CI)
Trimester 1a (first 6 weeks) (*n* = 1324)					
Abstinent	9.31 (9.08–9.54)	9.35 (9.12–9.59)	9.35 (9.11–9.59)	9.35 (9.11–9.59)	9.36 (9.12–9.6)
Low (≤7 drinks per week, up to 2 per occasion)	9.02 (8.71–9.34)	9 (8.69–9.32)	9 (8.69–9.32)	8.98 (8.66–9.29)	8.98 (8.66–9.29)
Moderate (≤7 drinks per week, > 2 to ≤4 per occasion)	8.82 (8.11–9.54)	8.72 (8.01–9.43)	8.72 (8–9.43)	8.73 (8.02–9.45)	8.71 (7.99–9.43)
Binge (≤7 drinks per week, > 4 per occasion)	9.08 (8.72–9.44)	9.04 (8.68–9.4)	9.04 (8.68–9.4)	9.06 (8.7–9.42)	9.07 (8.7–9.43)
Heavy (> 7 drinks per week, weekly or more)	9.33 (8.94–9.72)	9.31 (8.92–9.7)	9.33 (8.93–9.72)	9.33 (8.93–9.73)	9.31 (8.91–9.71)
Trimester 1b* (second 6 weeks) (*n* = 1227)					
Abstinent	9.23 (9.06–9.4)	9.24 (9.07–9.41)	9.24 (9.07–9.41)	9.24 (9.07–9.41)	9.25 (9.08–9.42)
Low (≤7 drinks per week, up to 2 per occasion)	9 (8.64–9.35)	8.93 (8.58–9.29)	8.94 (8.58–9.3)	8.93 (8.57–9.3)	8.92 (8.56–9.29)
Trimester 2* (*n* = 1259)					
Abstinent	9.16 (8.99–9.33)	9.15 (8.98–9.32)	9.15 (8.97–9.32)	9.15 (8.98–9.33)	9.16 (8.98–9.33)
Low (≤7 drinks per week, up to 2 per occasion)	9.15 (8.86–9.43)	9.18 (8.89–9.47)	9.18 (8.89–9.47)	9.17 (8.87–9.46)	9.15 (8.86–9.44)
Trimester 3* (*n* = 1270)					
Abstinent	9.22 (9.04–9.39)	9.19 (9.02–9.37)	9.19 (9.02–9.37)	9.2 (9.02–9.37)	9.21 (9.03–9.38)
Low (≤7 drinks per week, up to 2 per occasion)	9.09 (8.81–9.38)	9.16 (8.87–9.45)	9.16 (8.87–9.45)	9.15 (8.86–9.45)	9.13 (8.83–9.43)

Note: *Standard drink* = 10 g of alcohol. *Moderate, *Binge* and *Heavy* categories were not assessed after T1a due to infrequent reporting of these drinking patterns in the sample

^a^Adjusted for Mother-related background variables (Age at birth, Education, SEIFA, State of residence, Country of birth, Single parent household, Aboriginal and Torres Strait Islander status, Native language)

^b^Adjusted for Mother-related background variables + Substance use variables (Pregnancy smoked, Pregnancy illicit drugs)

^c^Adjusted for Mother-related background variables + Physical and psychological variables (Pregnancy Anxiety, IQ, Parity, BMI)

^d^Adjusted for all previous Mother-related variables + Infant-related variables (Gestational age)

As paternal factors have been associated with maternal drinking in pregnancy, a second series of regression analyses were conducted within a sub-sample of the women whose partners participated in the study (Additional file 1: Tables S3 and S4). Again, PAE was not significantly associated with GM development at 12-months (See Additional file 1: Section A for sensitivity analyses).

### Propensity score matching: low level exposure versus no exposure

Using propensity score matching, 308 abstinent mothers were matched to 312 low-level drinkers in Trimester 2. Results indicated that GM scores did not differ significantly between children born to drinkers (M = 9.20, SD = 2.85) and those born to abstainers (M = 8.82, SD = 2.52; t = − 1.75, *p* = 0.08).

The sample was then stratified into two levels of risk based on the propensity score matching (see Additional file 2: Section B). Highest risk of alcohol exposure was found to be related to factors including higher SES, higher education, older age, being of English speaking origin, tobacco use, and unplanned pregnancy. In the highest risk subgroup, 122 abstainers were matched to 195 drinkers. In the lowest risk subgroup, there were 186 abstinent women matched to 117 drinkers. Significant differences were only observed between drinkers and abstainers in the highest risk group (t = − 2.92, *p* = 0.004), with children born to low-level drinkers having higher GM scores (M = 9.20, SD = 2.85) compared to those born to abstainers (M = 8.82, SD = 2.52) (see Additional file 2: Section B Figure S1).

## Discussion

This study used unique, multi-wave data on 1324 infant offspring from a longitudinal pregnancy cohort to examine the association between PAE and infant GM development at 12-months. The study specifically addressed PAE *timing* (i.e., four time-points in pregnancy) and *dose* (i.e., low, binge, moderate and heavy PAE in T1a; and low-level PAE thereafter). Results showed that alcohol use was common in pregnancy, particularly in the first 6-weeks, when parents were unaware of their pregnancy. Thereafter, any drinking by pregnant women generally occurred at low-levels (≤7 standard drinks per week, up to 2 standard drinks per occasion). No significant differences in BSID-III GM scale scores were identified among infants of abstainers compared with infants whose mothers reported any alcohol consumption in pregnancy, before or after adjustment for potential confounders.

### Patterns of pregnancy drinking: mothers and partners

Pregnancy drinking was common in this cohort: 56.1% of pregnant women drank in Trimester one, 0–6 weeks (T1a); 23.6% in Trimester one, 7–12 weeks (T1b); 30.5% in Trimester two (T2); and, 29.6% in Trimester three (T3), respectively. Most women reported drinking at low-levels (average 22.5% across pregnancy). With the exception of T1a, few women reported moderate (2.3%), binge (1.7%) or heavy (1.3%) drinking in pregnancy. Whilst this is consistent with past research, greater specificity in this cohort on PAE timing and dose highlights two findings of public health import [36, 37]. First, rates of drinking prior to pregnancy awareness are close to twice that following awareness; and second, binge and heavy drinking both occur at high rates within the pre-awareness period: 15.9 and 14.0%, respectively. Taken together, the very earliest period of pregnancy may be one of greatest risk of exposure to alcohol.

### Characteristics of women drinking in pregnancy and their partners

Consistent with past research, pregnant women who consumed alcohol differed on socio-demographic characteristics compared to abstainers [36, 37]. Specifically, they were more likely to be older, tertiary educated, have moderate to high SEIFA scores (reflective of socio-economic advantage), be born in Australia or another English speaking country, and less likely to live in a multiple parent household. Other factors associated with pregnancy drinking included: smoking in pregnancy; reduced anxiety; higher estimated IQ; and lower odds of obesity. These results suggest pregnancy drinking is common among women from more affluent socio-demographic backgrounds, and among specific at-risk groups such as women who smoke cigarettes. Targeting these populations may result in more effective preventive intervention for pregnancy drinking.

With respect to partners, our results are consistent with the limited extant literature [38, 39]. Specifically, partners of pregnant women who drink were more likely to be from advantaged socio-economic backgrounds (higher SEIFA scores and educational attainment), and to also drink alcohol and smoke tobacco. These characteristics may affect offspring development via their influence within the familial environment (i.e., partner drinking increases the risk for maternal drinking) [40]. Few studies have accounted for these potential influences when examining associations between PAE and offspring development [38].

### PAE and infant GM development

The third aim was to determine whether GM development was impaired among infants exposed to PAE compared with infant offspring of abstainers. Potential background socio-demographic confounders were included in the adjusted analyses, along with other potential confounders (maternal substance use, physical and psychological factors; infant factors) associated with PAE exposure in the univariate analyses. Finally, to account for the potential role of partner factors in determining infant outcomes, we re-ran the models in a sub-set of the sample for whom partner data were available. We found no evidence to suggest that low PAE was associated with measurable impairments in infant GM development at 12-months. Moreover, at T1a (0–6 weeks), prior to pregnancy awareness for most women, neither moderate, binge nor heavy drinking predicted measurable GM impairment. In all models, and at all levels of adjustment, the pattern of results remained unchanged.

With regard to low-level exposure, the results are consistent with a small number of existing studies showing GM impairment was not linked to low PAE [20, 21]. There are a number of plausible explanations for this finding. First, that low PAE does not have a deleterious effect on early GM development. Alternatively, if harm does occur, the effects are likely to be very small. Our current measurement instruments, even though gold-standard, may not be sufficiently sensitive to detect such small GM deficits. Finally, it is well documented that infant development may fluctuate during infancy [41]. If low PAE does have harmful effects on GM development, these effects may be more readily detected in childhood as GM skills stabilise.

Importantly, when the sample was stratified by propensity score, the relationship between alcohol exposure and GM outcome was different relative to women’s baseline characteristics. In the highest risk subgroup, significant differences were observed between low-level drinkers and abstainers, with children born to low-level drinkers having higher GM scores compared to those born to abstainers. Consistent with other epidemiological findings [35], this result is suggestive of a potential interaction between PAE and other demographic and maternal risk factors in relation to offspring development, Further assessment of this interaction is recommended in samples with greater representation of women from low SES and high-risk backgrounds.

The finding that neither binge, moderate nor heavy PAE were linked to poorer GM development is inconsistent with a number of studies [2]. We did not examine potential harms after T1a due to the low frequency of these drinking patterns. It is possible that harmful drinking patterns later in pregnancy or persistent patterns of harmful drinking across the gestational window may be associated with GM impairment. It has been documented that there are sensitive gestational periods where risk for negative outcomes from teratogens may be heightened [42]. Differential impacts (relative to exposure timing) may explain inconsistency in the literature, and why it is difficult to determine a specific threshold at which PAE is harmful or safe for fetal development. Future work with greater representation of moderate, binge and heavy drinking patterns across the gestational period is needed, either through new cohorts with larger samples, targeted samples of moderate to higher-risk drinkers, and/or through potential data pooling/harmonisation [43].

### Limitations

There are a number of limitations. First, women with low SES backgrounds (and their partners) were underrepresented, and those who were included tended to be abstainers rather than drinkers. As such, this study may not have captured low SES and marginalised families, whose children may be most susceptible to harms relating to PAE [44]. When the sample was stratified by propensity score, the relationship between alcohol exposure and GM outcome was different for women with different patterns of baseline characteristics. Results should thus be interpreted with the caveat that associations between alcohol use and GM outcomes may show a different pattern among lower SES or high-risk populations. Targeted recruitment of these disadvantaged families may result in better representation of the effects of heavier PAE patterns across a range of demographics. Obtaining sufficient representation of heavier PAE post-awareness is difficult due to the reduction that occurs in drinking. Data pooling and/or harmonisation of existing cohorts might be one approach to address this issue [43]. Nevertheless, this study did have good representation of varying PAE within the first six weeks of pregnancy, particularly low PAE from T1a to birth, suggesting that the cohort was well-suited to the assessment of impacts of low PAE. Second, despite the use of the gold-standard, clinically administered BSID-III, GM skills can fluctuate in infancy [41]. Potential PAE effects not identified in this study may emerge as the cohort offspring develop and their capacities stabilise. Thus, follow-up of the cohort to assess development into childhood is important.

## Conclusion

This large-scale prospective study, with detailed assessment of PAE *timing* and *dose*, found no evidence to suggest that low PAE is associated with measurable impairment in infant GM development at 12-months. This result appears consistent with the limited available research [2]. Examination of higher exposure levels found that in T1a, prior to pregnancy awareness, neither moderate, binge, nor heavy PAE were associated with offspring GM impairment. Since most women either ceased or reduced their alcohol consumption following pregnancy awareness, this study was not able to examine the consequences of heavier drinking through pregnancy. We note that the present study focused on one developmental time-point; given the variability in GM development through infancy, it is possible that deleterious effects may be observed later in childhood. Further research is needed to examine potential PAE impacts on GM development through childhood.

## Additional files

Additional file 1: Section A **Table S1.** Patterns of alcohol use by partners across pregnancy. **Table S2.** Paternal factors associated with alcohol use by mothers. **Table S3.** Regression results for maternal alcohol use and infant gross motor outcomes; women with partner in study only. **Table S4.** Marginal means for maternal alcohol use and infant gross motor outcomes; women with partner in study only. (DOCX 24 kb)

Additional file 2: Section B Description of propensity score matching analysis. **Figure S1.** Infant gross motor score in low prenatal alcohol exposure vs. abstinent group, stratified by propensity score. (DOCX 20 kb)

## Abbreviations

BSID-III: The Bayely Scales of Infant Development, Third edition
GM: Gross motor
PAE: Prenatal alcohol exposure
T1a: Trimester one, 0–6 weeks
T1b: Trimester one, 7–12 weeks
T2: Trimester two, 13–27 weeks
T3: Trimester three, 28+ weeks
