# Correction to: Prenatal alcohol exposure and infant gross motor development: a prospective cohort study

**DOI:** 10.1186/s12887-019-1585-5

**Published:** 2019-07-04

**Authors:** Delyse Hutchinson, George J. Youssef, Clare McCormack, Judy Wilson, Steve Allsop, Jake Najman, Elizabeth Elliott, Lucinda Burns, Sue Jacobs, Ingrid Honan, Larissa Rossen, Hannah Fiedler, Samantha Teague, Joanne Ryan, Craig A. Olsson, Richard P. Mattick

**Affiliations:** 1 National Drug and Alcohol Research Centre, University of New South Wales, Sydney, Australia; 2 Faculty of Health, School of Psychology, Centre for Social and Early Emotional Development, Deakin University, Geelong, Victoria Australia; 3 Murdoch Children’s Research Institute, Centre for Adolescent Health, Royal Children’s Hospital, Melbourne, Australia; 4 Department of Paediatrics, University of Melbourne, Royal Children’s Hospital, Melbourne, Australia; 5 National Drug Research Institute, Curtin University, Perth, Australia; 6 Queensland Alcohol and Drug Research and Education Centre and Schools of Public Health and Social Science, University of Queensland, Brisbane, Australia; 7 Discipline of Child and Adolescent Health, Faculty of Medicine and Health, University of Sydney, Sydney, Australia; 8 Sydney Children´s Hospitals Network, Westmead, Sydney, Australia; 9 Department of Obstetrics, Royal Prince Alfred Hospital, Sydney, Australia; 10 School of Psychological Sciences, University of Melbourne, Melbourne, Victoria Australia

**Correction to: BMC Pediatr**

**https://doi.org/10.1186/s12887-019-1516-5**

Following publication of the original article [1], the authors opted to revise two paragraphs of the article text.

Firstly, they revised the first paragraph under subsection “**Characteristics associated with maternal drinking in pregnancy**”. Below is the updated version:

**Characteristics associated with maternal drinking in pregnancy**

Univariate tests compared whether abstainers and pregnancy drinkers (at any level) differed on background socio-demographics, other substance use, and physical and psychological factors (Table 3). The results show that, relative to abstainers, women who drank alcohol had greater odds of being older (e.g., 30–35 years, 1.97, 95% CI, 1.20–3.24); completing high school (2.61, 95% CI, 1.48–4.61); having moderate (2.29, 95% CI, 1.31–4.02) or high SES (4.42, 95% CI, 2.56–7.64); being born in an English speaking country (1.88, 95% CI, 1.33–2.66); and speaking English as their first language [2].34, 95% CI, 1.77–3.09); and lower odds of living in a single parent household (0.61, 95% CI, 0.39-0.95). Other factors associated with pregnancy drinking included: smoking in pregnancy (1.67, 95% CI, 1.18–2.36); higher estimated IQ (e.g., a score of 100–114, 3.02, 95% CI, 2.01–4.53); and lower anxiety (0.76, 95% CI, 0.57–0.99).

Secondly, they revised the first paragraph under subsection “**Characteristics of women drinking in pregnancy and their partners**”. Please see below:

**Characteristics of women drinking in pregnancy and their partners**

Consistent with past research, pregnant women who consumed alcohol differed on socio-demographic characteristics compared to abstainers [36, 37]. Specifically, they were more likely to be older, tertiary educated, have moderate to high SEIFA scores (reflective of socio-economic advantage), be born in Australia or another English speaking country, and be less likely to live in a single parent household. Other factors associated with pregnancy drinking included: smoking in pregnancy; higher estimated IQ; and lower levels of anxiety. These results suggest pregnancy drinking is common among women from more affluent socio-demographic backgrounds, and among specific at-risk groups, such as women who smoke cigarettes. Targeting these populations may result in more effective preventive intervention for pregnancy drinking.
