# Supplementary material for: Prenatal alcohol exposure and infant gross motor development: a prospective cohort study
Source: BMC Pediatr. 2019 May 14;19:149. doi: 10.1186/s12887-019-1516-5 (PMC6515673; doi:10.1186/s12887-019-1516-5)
Supplement: Supplementary file 1 — Section A Table S1. Patterns of alcohol use by partners across pregnancy. Table S2. Paternal factors associated with alcohol use by mothers. Table S3. Regression results for maternal alcohol use and infant gross motor outcomes; women with partner in study only. Table S4. Marginal means for maternal alcohol use and infant gross motor outcomes; women with partner in study only. (DOCX 24 kb) [file 12887_2019_1516_MOESM1_ESM.docx]

Supplementary Table 1

*Patterns of alcohol use by partners across pregnancy (N=755)*

|  | **Alcohol use category** | | | | |
| --- | --- | --- | --- | --- | --- |
|  | **Abstinent** | **Low**  (≤7 drinks per week, up to 2 per occasion) | **Moderate**  (<14 drinks per week, >2 to ≤4 per occasion) | **Binge**  (≤14 drinks per week, >4 per occasion) | **Heavy**  (>14 drinks per week, weekly or more) |
| **Trimester 3** |  |  |  |  |  |
| n (%) | **109 (14.4)** | **154 (20.3)** | **109 (14.4)** | **211 (27.9)** | **101 (13.3)** |
| Drinking days per week, M (SD) | 0 | 2.21 (2.07) | 1.48 (1.07) | 1.72 (1.30) | 4.44 (1.75) |
| Typical grams consumed per occasion, M (SD) | 0 | 14.74 (2.51) | 30.86 (3.11) | 50.08 (4.31) | 63.86 (38.93) |
| Typical grams consumed per week, M (SD) | 0 | 31.86 (29.60) | 45.27 (32.58) | 63.84 (35.88) | 23.95 (128.65) |

Note: (%) values do not sum to 100% due to missing data. *Standard drink* = 10 grams of alcohol. Antenatal alcohol use by partners was only assessed in Trimester 3.

Supplementary Table 2

*Paternal factors associated with alcohol use by mothers (pooled data, N=755)*

|  | **Abstainers**  **(n%)** | **Drinkers**  **(n%)** | | **Drinkers vs abstainers - Unadjusted OR**  **(95% CI)** | |
| --- | --- | --- | --- | --- | --- |
|  |  |  |  |  |  |
| Alcohol use |  |  | |  | |
| Abstinent | 77 (31.8) | 46 (8.9) | | Ref | |
| Low | 61 (25.3) | 108 (21.2) | | 2.99 (1.79-5.00)*** | |
| Mod | 34 (14) | 86 (16.8) | | 4.27 (2.40-7.63)*** | |
| Binge | 50 (20.7) | 181 (35.4) | | 6.08 (3.65-10.12)*** | |
| Heavy | 20 (8.1) | 91 (17.7) | | 7.78 (4.06-14.91)*** | |
| Age |  |  | |  | |
| ≤ 24 | 24 (9.9) | 21 (4.1) | | Ref | |
| 25-29 | 74 (30.6) | 139 (27) | | 2.11 (1.08-4.12)* | |
| 30-35 | 100 (41.1) | 255 (49.7) | | 2.89 (1.52-5.52)** | |
| ≥ 36 | 45 (18.4) | 98 (19.1) | | 2.48 (1.23-4.98)* | |
| Level of education |  |  | |  | |
| Less than Year 12 | 16 (6.7) | 34 (6.7) | | Ref | |
| Year 12 | 29 (11.9) | 61 (11.9) | | 1 (0.47-2.13) | |
| Certificate / Diploma | 57 (23.4) | 107 (20.9) | | 0.89 (0.45-1.76) | |
| Bachelor or higher | 141 (57.9) | 310 (60.5) | | 1.04 (0.55-1.96) | |
| Country of birth |  |  | |  | |
| Australia | 126 (52) | 293 (57.1) | | Ref | |
| Other English speaking | 42 (17.5) | 134 (26.2) | | 1.36 (0.9-2.07) | |
| NESB | 74 (30.5) | 85 (16.7) | | 0.5 (0.34-0.73)*** | |
| Aboriginal and Torres Strait Islander |  |  | |  | |
| No | 237 (97.5) | 506 (98.8) | | Ref | |
| Yes | 6 (2.5) | 6 (1.2) | | 0.47 (0.15-1.47) | |
| English First language |  |  | |  | |
| No | 76 (31.4) | 91 (17.8) | | Ref | |
| Yes | 166 (68.6) | 421 (82.2) | | 2.12 (1.43-3.15)*** | |
| Tobacco in pregnancy |  |  | |  | |
| No | 198 (81.5) | 419 (81.7) | | Ref | |
| Yes | 45 (18.5) | 94 (18.3) | | 0.99 (0.66-1.48) | |
| Illicit substances ever in pregnancy |  |  | |  | |
| No | 232 (95.7) | 475 (92.8) | | Ref | |
| Yes | 11 (4.3) | 37 (7.2) | | 1.71 (0.83-3.51) | |
| Anxiety |  |  | |  | |
| Normal | 228 (94.1) | 488 (95.2) | | Ref | |
| Elevated | 14 (5.9) | 24 (4.8) | | 0.79 (0.4-1.59) | |
| Stress |  |  | |  | |
| Normal | 220 (90.7) | 477 (93.1) | | Ref | |
| Elevated | 23 (9.3) | 35 (6.9) | | 0.72 (0.4-1.28) | |
| Depression |  |  | |  | |
| Normal | 217 (89.3) | 463 (90.4) | | Ref | |
| Elevated | 217 (89.3) | 463 (90.4) | | 0.89 (0.51-1.57) | |
| Victim of spousal abuse |  |  | |  | |
| No | 230 (94.7) | 475 (92.7) | | Ref | |
| Yes | 13 (5.3) | 38 (7.3) | | 1.43 (0.72-2.86) | |
|  |  |  | |  | |
| Estimated IQ (All partners) |  |  | |  | |
| ≤ 84 | 32 (13.3) | 44 (8.6) | | Ref | |
| 85-99 | 72 (29.7) | 143 (27.9) | | 1.46 (0.73-2.9) | |
| 100-114 | 86 (35.6) | 193 (37.6) | | 1.64 (0.85-3.14) | |
| ≥ 115 | 52 (21.5) | 133 (25.9) | | 1.87 (0.91-3.83) | |
| Estimated IQ (Native English speakers only n=572) |  |  | |  | |
| ≤ 84 | 17 (6.9) | 33 (6.5) | | Ref | |
| 85-99 | 61 (25.3) | 139 (27.1) | | 1.12 (0.38-3.29) | |
| 100-114 | 96 (39.4) | 194 (37.8) | | 1.01 (0.37-2.75) | |
| ≥ 115 | 69 (28.3) | 146 (28.5) | | 1.06 (0.38-2.95) | |
| Body Mass Index |  |  | |  | |
| Underweight | 0 (0) | 1 (0.2) | | N/A (zero cells) | |
| Normal weight | 98 (40.3) | 210 (40.9) | | Ref | |
| Overweight | 99 (40.8) | 229 (44.7) | | 1.08 (0.74-1.56) | |
| Obese | 46 (18.9) | 73 (14.2) | | 0.74 (0.45-1.23) | |
| **p*<0.05; ***p*<0.01, ****p*<0.001 |  | |  | |  |

Supplementary Table 3

*Regression results for maternal alcohol use and infant gross motor outcomes; women with partner in study only (pooled data, N=755)*

|  | **Unadjusted**  **b (95%CI)** | **Adjusted for maternal^a^**  **b (95%CI)** | **Adjusted for maternal^b^**  **b (95%CI)** | **Adjusted for maternal^c^**  **b (95%CI)** | **Adjusted for maternal + infant^d^**  **b (95%CI)** | **Adjusted for maternal + infant + partner^e^**  **b (95%CI)** | **Adjusted for maternal + infant + partner^f^**  **b (95%CI)** |
| --- | --- | --- | --- | --- | --- | --- | --- |
| **Trimester 1a (first 6 weeks) (n=755)** |  |  |  |  |  |  |  |
| Abstinent | Ref | Ref | Ref | Ref | Ref | Ref | Ref |
| Low | 0.07 (-0.46-0.61) | 0.01 (-0.53-0.55) | -0.01 (-0.55-0.53) | -0.05 (-0.6-0.49) | -0.08 (-0.62-0.47) | -0.04 (-0.6-0.51) | -0.05 (-0.61-0.51) |
| Moderate | 0.19 (-0.82-1.2) | -0.01 (-1.02-1.01) | -0.04 (-1.05-0.98) | -0.03 (-1.05-0.99) | -0.1 (-1.12-0.92) | 0 (-1.03-1.03) | 0.04 (-1-1.07) |
| Binge | -0.33 (-0.9-0.25) | -0.35 (-0.95-0.25) | -0.42 (-1.03-0.19) | -0.4 (-1.02-0.22) | -0.41 (-1.03-0.2) | -0.39 (-1.01-0.22) | -0.4 (-1.03-0.22) |
| Heavy | 0.01 (-0.61-0.63) | -0.15 (-0.78-0.48) | -0.2 (-0.84-0.43) | -0.19 (-0.84-0.46) | -0.22 (-0.87-0.42) | -0.18 (-0.83-0.47) | -0.27 (-0.94-0.39) |
| **Trimester 1b*** **(second 6 weeks) (n=710)** |  |  |  |  |  |  |  |
| Abstinent | Ref | Ref | Ref | Ref | Ref | Ref | Ref |
| Low | -0.14 (-0.68-0.39) | -0.28 (-0.83-0.26) | -0.29 (-0.84-0.25) | -0.33 (-0.88-0.22) | -0.34 (-0.89-0.21) | -0.31 (-0.87-0.24) | -0.36 (-0.92-0.21) |
| **Trimester 2*** **(n=724)** |  |  |  |  |  |  |  |
| Abstinent | Ref | Ref | Ref | Ref | Ref | Ref | Ref |
| Low | -0.06 (-0.5-0.38) | -0.08 (-0.53-0.38) | -0.08 (-0.53-0.38) | -0.09 (-0.55-0.37) | -0.12 (-0.58-0.34) | -0.08 (-0.55-0.38) | -0.12 (-0.59-0.34) |
| **Trimester 3*** **(n=724)** |  |  |  |  |  |  |  |
| Abstinent | Ref | Ref | Ref | Ref | Ref | Ref | Ref |
| Low | 0.06 (-0.37-0.5) | 0.06 (-0.39-0.52) | 0.06 (-0.4-0.52) | 0.02 (-0.45-0.49) | -0.02 (-0.49-0.45) | -0.01 (-0.49-0.46) | -0.07 (-0.55-0.41) |

Note: **Moderate*, *Binge* and *Heavy* categories were not assessed after T1a due to infrequent reporting of these drinking patterns in the sample.

^a^Adjusted for Mother-related background variables (Age at birth, Education, SEIFA, State of residence, Country of birth, Single parent household, Aboriginal and Torres Strait Islander status, Native language)

^b^Adjusted for Mother-related background variables + Substance use variables (Pregnancy smoked, Pregnancy illicit drugs)

^c^Adjusted for Mother-related background variables + Physical and psychological variables (Pregnancy Anxiety, IQ, Parity, BMI)

^d^Adjusted for all previous Mother-related variables + Infant-related variables (Gestational age)

^e^Adjusted for all previous Mother and Infant-related variables + Partner-related variables (Age at birth, Education, Country of birth, Aboriginal and Torres Strait Islander status, Native language)

^f^Adjusted for all previous Mother-, Infant- and Partner-related variables + Partner drank

Supplementary Table 4

*Marginal means for maternal alcohol use and infant gross motor outcomes; women with partner in study only (pooled data, N=755)*

|  | **Unadjusted**  **M (95%CI)** | **Adjusted for maternal^1^**  **M (95%CI)** | **Adjusted for maternal^2^**  **M (95%CI)** | **Adjusted for maternal^3^**  **M (95%CI)** | **Adjusted for maternal + infant^4^**  **M (95%CI)** | **Adjusted for maternal + infant + partner^5^**  **M (95%CI)** | **Adjusted for maternal + infant + partner^6^**  **M (95%CI)** |
| --- | --- | --- | --- | --- | --- | --- | --- |
| **Trimester 1a (first 6 weeks) (n=755)** |  |  |  |  |  |  |  |
| Abstinent | 9.28 (8.96-9.6) | 9.33 (9-9.65) | 9.35 (9.03-9.68) | 9.36 (9.03-9.69) | 9.37 (9.05-9.7) | 9.35 (9.02-9.68) | 9.37 (9.03-9.7) |
| Low | 9.35 (8.93-9.77) | 9.34 (8.92-9.75) | 9.34 (8.93-9.76) | 9.3 (8.88-9.72) | 9.3 (8.88-9.72) | 9.31 (8.89-9.73) | 9.32 (8.89-9.74) |
| Moderate | 9.47 (8.51-10.42) | 9.32 (8.37-10.28) | 9.32 (8.36-10.27) | 9.33 (8.37-10.28) | 9.28 (8.32-10.23) | 9.35 (8.39-10.31) | 9.41 (8.44-10.37) |
| Binge | 8.95 (8.48-9.43) | 8.98 (8.5-9.46) | 8.93 (8.45-9.42) | 8.96 (8.47-9.45) | 8.96 (8.47-9.45) | 8.96 (8.47-9.45) | 8.96 (8.47-9.46) |
| Heavy | 9.29 (8.75-9.82) | 9.18 (8.64-9.71) | 9.15 (8.62-9.69) | 9.17 (8.63-9.71) | 9.15 (8.61-9.69) | 9.17 (8.62-9.72) | 9.09 (8.53-9.65) |
| **Trimester 1b*** **(second 6 weeks) (n=710)** |  |  |  |  |  |  |  |
| Abstinent | 9.28 (9.05-9.51) | 9.31 (9.08-9.54) | 9.31 (9.08-9.54) | 9.32 (9.09-9.55) | 9.32 (9.09-9.55) | 9.32 (9.09-9.55) | 9.33 (9.09-9.56) |
| Low | 9.14 (8.67-9.61) | 9.03 (8.55-9.5) | 9.02 (8.54-9.5) | 8.99 (8.51-9.47) | 8.98 (8.5-9.46) | 9 (8.52-9.49) | 8.97 (8.48-9.46) |
| **Trimester 2*** **(n=724)** |  |  |  |  |  |  |  |
| Abstinent | 9.22 (8.99-9.46) | 9.23 (8.99-9.46) | 9.23 (8.99-9.46) | 9.23 (9-9.47) | 9.24 (9.01-9.47) | 9.23 (8.99-9.46) | 9.24 (9.01-9.48) |
| Low | 9.16 (8.79-9.53) | 9.15 (8.77-9.53) | 9.15 (8.77-9.53) | 9.14 (8.76-9.52) | 9.12 (8.73-9.5) | 9.14 (8.76-9.53) | 9.12 (8.73-9.5) |
| **Trimester 3*** **(n=724)** |  |  |  |  |  |  |  |
| Abstinent | 9.24 (9-9.47) | 9.24 (9-9.47) | 9.24 (9-9.47) | 9.25 (9.01-9.48) | 9.26 (9.02-9.49) | 9.26 (9.02-9.49) | 9.27 (9.04-9.51) |
| Low | 9.3 (8.93-9.67) | 9.3 (8.92-9.68) | 9.3 (8.91-9.68) | 9.27 (8.88-9.66) | 9.24 (8.85-9.63) | 9.24 (8.85-9.64) | 9.2 (8.8-9.6) |

Note: Bold represents p<.05. **Moderate*, *Binge* and *Heavy* categories were not assessed after T1a due to infrequent reporting of these drinking patterns in the sample.

^1^Adjusted for Mother-related background variables (Age at birth, Education, SEIFA, State of residence, Country of birth, Single parent household, Aboriginal and Torres Strait Islander status, Native language)

^2^Adjusted for Mother-related background variables + Substance use variables (Pregnancy smoked, Pregnancy illicit drugs)

^3^Adjusted for Mother-related background variables + Physical and psychological variables (Pregnancy Anxiety, IQ, Parity, BMI)

^4^Adjusted for all previous Mother-related variables + Infant-related variables (Gestational age)

^5^Adjusted for all previous Mother and Infant-related variables + Partner-related variables (Age at birth, Education, Country of birth, Aboriginal and Torres Strait Islander status, Native language)

^6^Adjusted for all previous Mother-, Infant- and Partner-related variables + Partner drank
