# Supplementary material for: Prenatal alcohol exposure and infant gross motor development: a prospective cohort study
Source: BMC Pediatr. 2019 May 14;19:149. doi: 10.1186/s12887-019-1516-5 (PMC6515673; doi:10.1186/s12887-019-1516-5)
Supplement: Supplementary file 2 — Section B Description of propensity score matching analysis. Figure S1. Infant gross motor score in low prenatal alcohol exposure vs. abstinent group, stratified by propensity score. (DOCX 20 kb) [file 12887_2019_1516_MOESM2_ESM.docx]

# Supplementary Section B

Propensity Score Matching is used when randomisation to conditions is not possible to ensure baseline characteristics are consistent between groups, and that any difference between groups is attributable to ‘treatment’ or exposure effects [52, 53].

A score was calculated that reflects the propensity of a woman to consume alcohol at low-levels during Trimester 2 (T2) based on a logistic regression analysis (i.e., with a binary outcome indicating low-level drinking status at T2). T2 was selected as this is the time point at which low-level prenatal alcohol exposure was most prevalent. This score was used to pair each participant in the drinking group with a close match in the abstaining group. Variables used to define the propensity score were selected from baseline characteristics and entered into the logistic regression analyses. Matching was done without replacement, and priority was given to exact matches. Match tolerance was conservatively set at 0.1 to minimise mean squared error [54].

The propensity scores were then used in analysis. First, the samples of women who drank at low-levels in T2 and abstainers (which were now matched using the propensity scores) were directly compared on GM functioning using independent samples T-tests. Next, to examine whether the effect of PAE on GM outcomes may differ depending on an individual’s initial risk for endorsing low-level alcohol exposure during pregnancy (i.e., based on their baseline characteristics, which were used to derive the propensity scores), samples were stratified into two separate groups indicating higher or lower risk of low-alcohol exposure (versus abstinence). T-tests were then conducted which compared abstainers to drinkers on GM functioning, separately for groups of higher or lower risk based on their propensity score.

Figure 1:

*Infant gross motor score in low prenatal alcohol exposure vs. abstinent group, stratified by propensity score*

Note: *p<.01

# References

52. Austin MP, Grant K, McMahon C, Reilly N. Prenatal anxiety disorder, maternal sensitivity and neurodevelopmental outcomes in infants. Arch Womens Ment Health. 2011;14:S7–8. 741

53. Rosenbaum PR, Rubin DB. Constructing a control group using multivariate matched sampling methods that incorporate the propensity score. Am Stat. 1985;39:33–8. 744

54. Austin PC. A critical appraisal of propensity-score matching in the medical literature between 1996 and 2003. Stat Med. 2008;27:2037–49.
